# Insights into the effects of apelin-13 on renal function and NHE3 activity following ischemia/reperfusion-induced acute kidney injury

**DOI:** 10.3389/fphys.2025.1544274

**Published:** 2025-03-19

**Authors:** Guilherme Lopes-Gonçalves, Juliana Martins Costa-Pessoa, Mariana Charleaux de Ponte, Heitor Macedo Braz, Maria Oliveira-Souza

**Affiliations:** ^1^ Laboratory of Renal Physiology, Department of Physiology and Biophysics, Institute of Biomedical Sciences, University of Sao Paulo, Sao Paulo, Brazil; ^2^ Laboratory of Cellular and Molecular Bases of Renal Physiology, Department of Physiology and Biophysics, Institute of Biomedical Sciences, University of Sao Paulo, Sao Paulo, Brazil

**Keywords:** ischemia-induced acute kidney injury, apelin-13, tubular injury, tubular proliferation, NHE3

## Abstract

**Introduction:**

Acute kidney injury (AKI) is a clinical syndrome characterized by rapid decline in renal function with varying severity. In this context, tubular function is impaired in ischemia-induced AKI. Although there are no effective therapies for AKI, many compounds have been reported to reduce kidney injury, such as apelin-13. Considering the relevance of proximal tubular cells in maintaining fluid and electrolyte homeostasis, the effects of apelin-13 on tubular injury or sodium proximal transport remain unclear. Thus, the present study aims to evaluate the effects of exogenous administration of apelin-13 in the renal ischemia/reperfusion (I/R) model, with particular focus on renal function, injury markers, and tubular proliferation.

**Methods:**

Male C57BL/6 mice were initially treated with a vehicle or high dose of apelin-13 (200 μg/kg/day) and subjected to kidney bilateral ischemia procedure for 30 min or a sham surgery. The mice were euthanized by exsanguination 2 d after the ischemic procedure. Then, the renal function was assessed through the plasma urea level and creatinine clearance. Tubular injury was evaluated by hematoxylin and eosin staining. Kidney injury molecule 1 (KIM-1), neutrophil gelatinase-associated lipocalin (NGAL), megalin, Ki67, and phospho ERK 1/2 (Thr202/Tyr204) were evaluated through immunohistochemical or immunoblotting experiments. Moreover, the murine proximal tubular cells (TKPTS) were treated with apelin-13 (100 nM) to evaluate the activity of the Na^+^/H^+^ exchanger isoform 3 (NHE3) via intracellular pH measurements.

**Results:**

Initial administration of apelin-13 did not improve tubular injury, creatinine clearance, or plasma urea level after renal I/R. Moreover, KIM-1 and NGAL markers were markedly increased after renal I/R and were not reduced in the apelin-13 + I/R group. Furthermore, megalin downregulation by renal I/R was not prevented by apelin-13. Interestingly, apelin-13 worsened the renal responses to tubular proliferation after renal I/R as Ki67 and phosphorylation of ERK/1/2 (Thr202/Tyr204) were sharply reduced in the apelin-13 + I/R group. *In vitro* experiments also demonstrated that apelin-13 inhibited NHE3 activity in murine proximal tubular cells.

**Conclusion:**

The overall findings suggest that apelin-13 suppresses tubular proliferation and potentially impairs the adaptive response to renal I/R injury, thereby highlighting its relevance in ischemia-induced AKI.

## Highlights

• Exogenous apelin-13 administration does not attenuate tubular injury after renal ischemia/reperfusion.
•Apelin-13 impairs tubular proliferation after renal ischemia/reperfusion.
•Apelin-13 decreases the NHE3 activity of murine proximal tubular cells *in vitro.*

## Introduction

Acute kidney injury (AKI) is a clinical syndrome characterized by the rapid deterioration of kidney function along with structural damage (Kellum et al., 2021; Makris and Spanou, 2016). In clinical practice, AKI is a major concern whose incidence ranges from 5% to 20% among hospitalized patients (Han and Lee, 2019). Delayed diagnosis of AKI can result in higher risk of cardiovascular complications, development of chronic kidney disease (CKD) or end-stage renal disease (ESRD), and increased mortality rates (Pickkers et al., 2021). In this context, ischemia-induced AKI is a common subtype caused by obstruction of renal blood flow followed by reperfusion (Munshi et al., 2011). The pathophysiology of ischemia-induced AKI involves inflammation, apoptosis, cell-cycle arrest, and immune responses, with many local and soluble factors interacting with the renal tissues (Sharfuddin and Molitoris, 2011; Bonventre and Yang, 2011). Because of this complexity, it is challenging to find specific clinical therapies to prevent AKI (Guo et al., 2023).

Tubular epithelial cells are highly susceptible to injury during ischemia-induced AKI, with loss of the brush border and impaired cell polarity being the initial features of damage (Bonventre and Yang, 2011). Low adenosine triphosphate (ATP) availability leads to metabolic reprogramming of the proximal tubular cells (PTCs), which then upregulates glycolysis and reduces fatty acid oxidation (Li et al., 2021). These events are followed by mitochondrial injury, which significantly increases the generation of reactive oxygen species (ROS), leading to oxidative stress and DNA damage as well as predisposing the PTCs to apoptosis or detachment after reperfusion (Chen et al., 2024). Furthermore, injured PTCs can interrupt cellular mitosis, leading to epithelial–mesenchymal transitions and senescence-associated secretory phenotype, thereby increasing the release of interleukins (IL-1, 4, 6, and 18), tumor necrosis factor alpha (TNF-α), and chemokines such as monocyte chemoattractant protein-1 (MCP-1). This establishes an integrated network of inflammation and immune response (Zhang et al., 2024; Liu et al., 2017a).

Given the consequent reductions in renal blood flow and glomerular filtration rate in ischemia-induced AKI, the cellular dynamics and activities of the membrane transporters are impaired owing to tubular injury (Vallon, 2016). Since the PTC transporters regulate body and fluid homeostasis, they are involved in the immediate response of ischemia-induced AKI, acting as sensors (Vallon, 2016) to trigger or connect the initial signaling pathways that sustain tubular injury. Among the sodium transporters, solute carrier (SLC) proteins, such as the Na^+^/H^+^ exchanger isoform 3 (NHE3), are highly expressed at the brush border of the PTCs, and the basal activity of NHE3 is essential for Na^+^ and HCO_3_^−^ reabsorption, acid excretion, extracellular volume balance, and overall renal function in the healthy state (Fenton et al., 2017; Li et al., 2013). Evidence indicates that the depletion of intracellular ATP caused by mitochondrial injury during ischemia-induced AKI leads to reduced NHE3 expression and activity, which may account for the acid–base imbalances and natriuresis observed upon reperfusion (Di Sole et al., 2011; Kwon et al., 2000).

Cell proliferation and regeneration are fundamental processes in the recovery of PTCs following an ischemia-induced AKI episode (Li et al., 2024b; Bonventre and Yang, 2011). These reparative mechanisms involve multiple signaling cascades, with particular emphasis on the mitogen-activated protein kinases (MAPKs) (Luo et al., 2016). Notably, the extracellular-signal-regulated kinase 1/2 (ERK 1/2) pathway facilitates proliferation of PTCs after injury (Zhou et al., 2023; Hu et al., 2021a; Jang et al., 2013). Although effective treatments for AKI are still lacking in clinical practice (Gameiro et al., 2020), numerous compounds have been investigated for their potential to prevent this condition. Among these, the peptide apelin has emerged as a promising candidate for reducing kidney injury (Patil et al., 2024; de Oliveira et al., 2022; Chapman et al., 2021).

The apelinergic system comprises the peptide ligand apelin, ELABELA, and its G-protein-coupled receptor APLNR (Li et al., 2022; Guan et al., 2021; Chapman et al., 2021). Apelin is an adipokine that is broadly expressed in the entire body and has various regulatory functions, including glucose homeostasis, lipid metabolism, and water balance (Hu et al., 2021b). Apelin plasma concentration in healthy humans range from nanograms to picograms per milliliter (Bilgiç et al., 2016; Urwyler et al., 2016; Zhen et al., 2013). Apelin-13 is the most extensively studied isoform that is known for regulating glomerular hemodynamics and water balance in the kidneys (Girault-Sotias et al., 2021; Hus-Citharel et al., 2008). Apelin-13 has demonstrated promising results for mitigating certain aspects of kidney injury, such as mitochondrial dysfunction, autophagy impairment, and activation of profibrotic factors (Zheng et al., 2025; Liu et al., 2023; Topcu et al., 2023; Wang et al., 2017; Chen et al., 2015); however, the tubular mechanisms involved and potential adverse effects are largely undefined. Moreover, knowing that the kidney ischemia/reperfusion (I/R) mouse model can induce tubular damage comparable to ischemia-induced AKI in humans (Wei and Dong, 2012) and that PTCs are relevant for maintaining fluid homeostasis, the actions of apelin-13 on tubular injury or sodium proximal tubular transport need to be clarified.

Therefore, the present study aims to evaluate the effects of exogenous administration of apelin-13 in the renal I/R model, with a particular focus on renal function, injury markers, tubular proliferation, and regulation of NHE3 activity. We found that APLNR was induced in the renal tubular cells of the ischemia-induced AKI model, whereas initial/prior apelin-13 administration affected tubular proliferation after renal I/R. In addition, apelin-13 or I/R decreases NHE3 activity in the PTCs *in vitro*.

## Materials and methods

### Kidney I/R model

The animal study was conducted in accordance with the Animal Research: Reporting of *In Vivo* Experiments (ARRIVE) guidelines. All animal procedures were approved by the Institutional Animal Care and Use Committee of the Institute of Biomedical Sciences at the University of Sao Paulo, Brazil (CEUA-ICB/USP, protocol no. 2717131222). As described in a previous work (de Ponte et al., 2021), male C57BL/6 mice aged 8 weeks were acquired from the animal care facility of the School of Medicine, University of Sao Paulo, and housed in the experimental animal care facility of the Department of Physiology and Biophysics, Institute of Biomedical Sciences, University of Sao Paulo, Brazil. All animals were housed under standard conditions (22°C, 12/12-h light/dark cycles, 60% relative humidity, standard mice chow feed, and *ad libitum* water) for 1 week. As described in Supplementary Figure S1, the mice were randomly distributed into four groups for the experiments: vehicle + sham (n = 7), vehicle + I/R (n = 7), apelin-13 + sham (n = 6), and apelin-13 + I/R (n = 7). The animals were initially treated with either the vehicle (1× phosphate-buffered saline (PBS), pH 7.2) or a high dose of apelin-13 (200 μg/kg/day; purity ≥95%, no. 13523, Cayman Chemicals, Ann Arbor, MI, United States) (Li et al., 2018) in two daily intraperitoneal administrations (100 μg/kg every 12 h) for 5 days and subjected to the kidney bilateral ischemia procedure for 30 min or sham surgery on the fourth day. On the fifth day, the mice were individually placed in metabolic cages (Tecniplast, Buguggiate, VA, Italy) for 24 h urine collection. On the sixth day, all mice were maintained under inhalation anesthesia using 5% isoflurane. Blood samples were then collected from the heart, and the kidneys were removed. For each mouse, the right kidney was harvested for histological and protein analyses, while the left kidney was rapidly frozen in liquid nitrogen for RNA extraction. The mice were euthanized by exsanguination 2 days after the ischemia induction procedure.

### Kidney function analysis

As described in a previous work (Lopes-Gonçalves et al., 2023; de Ponte et al., 2021), urea and creatinine levels were analyzed using colorimetric assay kits (Labtest, Lagoa Santa, MG, Brazil) according to the manufacturer’s instructions.

### Urine albumin analysis

As described previously (Lopes-Gonçalves et al., 2023; de Ponte et al., 2021), 24-h urine aliquots containing 2.5 µg of creatinine were subjected to SDS-PAGE (10%), and albumin was identified using the SilverQuest Silver Staining kit (Sigma-Aldrich, St. Louis, MO, United States) according to the manufacturer’s instructions. The albumin bands were quantified in ImageJ software (National Institutes of Health, Bethesda, MD, United States).

### Tubular injury score

Based on minor modifications to a previously described procedure (de Ponte et al., 2021), the tubular injury score was evaluated from hematoxylin and eosin staining of 4-µm-thick kidney sections. Fifteen cortical or cortical–medullary areas were classified at 20× according to the following criteria: tubular swelling, cell and brush border loss, necrotic or proteinuric casts. Tubular injury was then graded on the following scale: grade 0, normal; grade 1, up to 25%; grade 2, 26%–50%; grade 3, 51%–75%; grade 4, >75%. A double-blinded analysis was also performed.

### Immunoblotting

Based on minor modifications to a previously described method (Lopes-Gonçalves et al., 2023), proteins from the frozen kidney tissues or fresh cultured cells were extracted using ice-cold 1× PBS (pH 7.4) enriched with protease and phosphatase inhibitors (Sigma-Aldrich). Immunoblotting was then performed on aliquots containing 30 μg/lane of proteins resolved on 10% SDS-PAGE. The proteins were wet transferred to 0.45-μm polyvinylidene fluoride membranes (Cytiva, Marlborough, MA, United States) and incubated for 1 h in a blocking solution containing 1× TRIS-buffered saline (TBS), 5% skimmed milk, and 0.1% Tween 20 at pH 7.4. Subsequently, the primary antibodies were diluted in the blocking solution and applied to the membranes before overnight incubation at 4°C: anti-Na^+^/H^+^ exchanger isoform 1 (NHE1; ab24018, 1:2000, Abcam, Cambridge, UK); anti-Na^+^/H^+^ exchanger isoform 3 (NHE3; ab307365, 1:1000, Abcam); anti-APLNR (711101, 1:500, Invitrogen, Carlsbad, CA, United States); anti-phospho ERK 1/2 (Thr202/Tyr204) (#4370, 1:2500, Cell Signaling Technology, Inc., Danvers, MA, United States); anti-ERK 1/2 (#06-182, 1:4000, Upstate Biotechnology, Lake Placid, NY, United States); anti-GAPDH (#2118, 1:2000, Cell Signaling). The following secondary antibodies were also used: peroxidase goat anti-rabbit IgG (111-035-003, 1:10000, Jackson ImmunoResearch Laboratories, Baltimore, MD, United States) and peroxidase goat anti-mouse IgG (115-035-003, 1:10000, Jackson ImmunoResearch Laboratories). The protein expressions were quantified using ImageJ software in terms of the phosphorylated/total protein ratio and represented as fold change with respect to the control group.

### Immunohistochemistry

As described previously (de Ponte et al., 2024; Lopes-Gonçalves et al., 2023; Lins et al., 2021; de Araújo et al., 2020), 4-µm-thick kidney sections were deparaffinized in xylene and rehydrated in a graded ethanol series ending in running tap water. The immunohistochemical (IHC) staining was analyzed with an Eclipse 80i microscope (Nikon, Tokyo, Japan) equipped with ×20 and ×40 plane objectives within NIS-Elements Basic Research software (Nikon). As described in the Supplementary Material, ImageJ software was used to open and quantify the files. The following antibodies were used in this step: anti-KIM-1 (AF 1817, 1:400, R&D Systems, Minneapolis, MN, United States); anti-megalin (sc16478, 1:250, Santa Cruz Biotechnology, Dallas, TX, United States); anti-APLNR (sc517300, 1:500, Santa Cruz Biotechnology); anti-Ki67 (ab16667, 1:200, Abcam); anti-phospho ERK 1/2 (Thr202/Tyr204) (#4370, 1:250, Cell Signaling); anti-NGAL (AF 1857, 1:500, R&D Systems); anti-NHE3 (ab307365, 1:100, Abcam).

### RNA extraction and quantitative real-time polymerase chain reaction (qRT-PCR)

As described in previous works (de Ponte et al., 2024; Lins et al., 2021; de Ponte et al., 2021), RNA was obtained from the whole kidney tissue sprayed with liquid nitrogen and resuspended in TRIzol (Invitrogen). RNA extraction was then performed using the Illustra RNAspin Mini RNA isolation kit (GE Healthcare, Chicago, IL, United States) according to the manufacturer’s instructions. Then, 2,000 ng of the total RNA was used to obtain cDNA using the High-Capacity cDNA Reverse Transcription Kit (Applied Biosystems, Waltham, MA, United States); qRT-PCR was then performed using a StepOne Real-Time PCR System (Applied Biosystems) along with predesigned TaqMan Gene Expression Assays (Applied Biosystems): Apelin (*Apln*) Mm00443562_m1; glyceraldehyde-3-phosphate dehydrogenase (*Gapdh*; reference gene) Mm99999915_g1. All qRT-PCR steps were performed in duplicate, and the samples were analyzed by the 2^−ΔΔCT^ method. The results were normalized to *Gapdh* expression and shown as units relative to the control group.

### Cell culture

Mouse proximal tubular epithelial cells (TKPTS) from the CRL-3361 line (American Type Culture Collection, Manassas, VA, United States) were cultured in DMEM/F-12 (1:1) medium supplemented with 7% fetal bovine serum (FBS), 5.6 μg/mL of insulin (I9278, Sigma-Aldrich), 100 IU/mL of penicillin, and 100 μg/mL of streptomycin in a humidified incubator (5% CO_2_, 37°C). The cells were grown in six-well plates until 70%–80% confluent, and passages 2–10 were used. As described in Supplementary Figure S1, the cells were treated with antimycin A (5 μM, Sigma-Aldrich) diluted in DMEM flex medium without glucose, pyruvate, or amino acids (A2493901, Thermo Fisher, Waltham, MA, United States) for 20 min to induce ischemia through ATP depletion. After washing once with 1× PBS, reperfusion was induced with the basal medium for 1 h. The cells were then treated with 100 nM of apelin-13 (Cayman Chemicals) for 24 h.

### Intracellular pH measurement

TKPTS cells were grown to 70%–80% confluence on glass coverslips. As described previously (Cardoso et al., 2018; Costa-Pessoa et al., 2013; Eguti et al., 2010; Thieme et al., 2008), the cells were fluorescently labeled with 2′,7′-bis-(2-carboxyethyl)-5-(and-6)-carboxyfluorescein acetoxymethyl ester (5 μM, BCECF-AM, Invitrogen) probe in ringer control solution containing Na^+^ [138 mM of NaCl, 5 mM of KCl, 1 mM of MgCl_2_, 0.8 mM of NaH_2_PO_4_, 0.83 mM of Na_2_HPO_4_, 1.8 mM of CaCl_2_, 8 mM of HEPES, 5 mM of glucose, pH 7.4] and subsequently rinsed before being placed in a thermoregulated chamber on an inverted epifluorescence microscope (Nikon). During the experiments, the cells were excited at wavelengths specific to BCECF-AM (495 nm and 440 nm), while the emissions were measured at 450 nm every 5 s. The ratio of dual excitation to single emission was calculated and converted to an intracellular pH (pHi) value using the high K^+^/nigericin solution [20 mM of NaCl, 130 mM of KCl, 1 mM of MgCl_2_, 1 mM of CaCl_2_, 5 mM of HEPES] containing 10 μM of nigericin adjusted to various pH values, as described previously (Costa-Pessoa et al., 2013; Thieme et al., 2008). The control, ischemic, and apelin-13-treated cells were then subjected to pHi recovery analysis, and the pHi recovery of the cells was examined using the NH_4_Cl prepulse technique (Boron and De Weer, 1976) [125 mM of NaCl, 5 mM of KCl, 1 mM of MgCl_2_, 0.8 mM of NaH_2_PO_4_, 0.83 mM of Na_2_HPO_4_, 1 mM of CaCl_2_, 8 mM of HEPES, 5 mM of glucose, 20 mM of NH_4_Cl, pH 8.0]. Sodium-independent pHi recovery was induced by perfusing the cells with a sodium-free ringer solution containing N-methyl-D-glucamine (NMDG) [138 mM of NMDG, 5 mM of KCl, 1 mM of MgCl_2_, 0.8 mM of NaH_2_PO_4_, 0.83 mM of Na_2_HPO_4_, 1.8 mM of CaCl_2_, 8 mM of HEPES, 5 mM of glucose, pH 7.4]. Sodium-dependent pHi recovery was induced by perfusing the cells with the ringer control sodium solution. All steps of pHi recovery for the apelin-13 treatment were performed using solutions containing 100 nM of apelin-13 (Cayman Chemicals). To evaluate the NHE3 contribution to the pHi recovery rate, the cells were treated with 10 µM of S3226 (Sigma-Aldrich) or 10 µM of cariporide (Santa Cruz Biotechnology) during the pHi recovery phase. To evaluate the NHE1 contribution to pHi recovery rate, the cells were treated with 1 nM of cariporide (Santa Cruz Biotechnology) during the pHi recovery phase. For all experiments, the initial pHi recovery rates were calculated (dpHi/dt, pH units/min) during the first 2 min of the recovery phase via linear regression analysis.

### Statistical analysis

Statistical analysis was performed using GraphPad Prism version 10.1 (GraphPad Software Inc., San Diego, CA, United States). For comparison between two groups, we used a two-tailed parametric unpaired t-test with Welch’s correction. The Shapiro–Wilk test was used to consider normality of the data. Comparisons between three or more groups were made using one-way or two-way ANOVA, followed by Bonferroni or Tukey multiple comparisons, respectively. All data were expressed as mean ± standard deviation (SD), and *p* < 0.05 was considered to be significant. The exact *p*-values are also presented in the graphs.

## Results

### Renal I/R upregulates apelin receptor expression and reduces apelin gene expression

**FIGURE 1:**
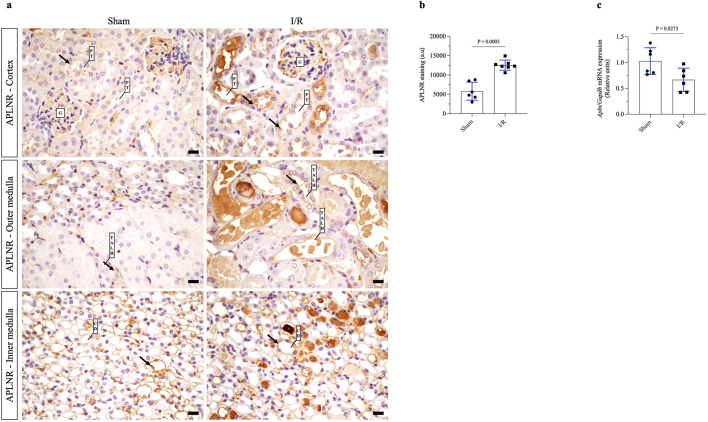
Renal ischemia/reperfusion (I/R) upregulates apelin receptor expression in kidney tissue and decreases apelin gene expression. **(A)** Representative immunohistochemistry from 40× micrographs (4 µm thick) and **(B)** graphical presentation showing that the apelin receptor (APLNR) was significantly increased in the renal I/R mice group. The black arrows indicate positive staining for APLNR. **(C)** Apelin mRNA expression (*Apln*) in whole kidney tissue from sham or I/R mice was analyzed by quantitative PCR. The results were normalized to *Gapdh* expression (endogenous gene) and are shown as relative units compared to the sham group. Statistical analysis was performed as *t*-test with Welch correction. The *p*-value is inserted in the graph. Sham mice (filled circles); I/R, ischemia/reperfusion mice (filled squares); G, glomerulus; PT, proximal tubule; TALH, thick ascending loop of Henle; CD, collecting duct; SD, standard deviation. Scale bar: 10 µm.

In the first set of *in vivo* experiments, we verified the staining of APLNR as well as expression of apelin mRNA in the renal tissues of sham or renal I/R mice. As shown in Figures 1A, B and Table 1, broad APLNR expressions were observed along the nephrons of the sham mice, while renal I/R induced a significant increase in the IHC staining of APLNR in the proximal tubules, thick ascending loop of Henle, and collecting ducts of the I/R mice compared to the sham group. Furthermore, renal I/R significantly decreased apelin mRNA expression compared to the sham group (Figure 1C; Table 1).

**TABLE 1:** Statistical data of animal experimentation.

Parameter	Sham (n)	I/R (n)
APLNR staining, a.u	5,824 ± 2,340 (6)	12,549 ± 1,305^***^ (7)
Apln mRNA expression, relative units	1.03 ± 0.257 (6)	0.666 ± 0.224^*^ (6)

The data were analyzed using t-test with Welch correction. ^*^ P < 0.05 versus sham, ^***^ P < 0.001 versus sham. The values are shown as mean ± SD. The number of animals (n) per group is indicated within parentheses. A.u, arbitrary units.

### Exogenous apelin-13 administration sustains kidney injury after renal I/R

**FIGURE 2:**
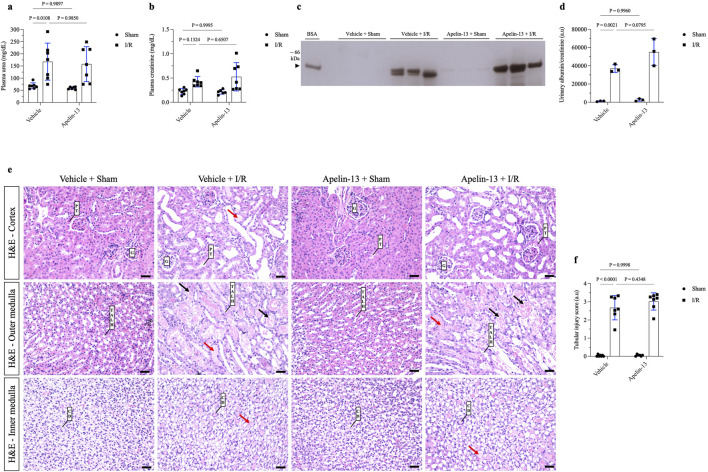
Prior administration of apelin-13 did not improve tubular morphology or kidney function after renal I/R. **(A)** Plasma urea analysis. **(B)** Plasma creatinine analysis. **(C)** Representative 10% SDS-PAGE and **(D)** graphical presentation showing the abundance of urinary albumin, which was not attenuated by apelin-13 administration. A bovine serum albumin (BSA, 0.2 mg/mL) standard was loaded on the adjacent lane (left). The cropped gel is displayed, and the full-length gel is included in Supplementary Figure S2. **(E)** Representative 20× micrographs of hematoxylin–eosin staining of kidney sections (4 µm thick) and **(F)** graphical presentation showing that prior administration of exogenous apelin-13 sustained tubular injury in mice subjected to renal I/R. The red arrows show protein casts inside the tubules, and the black arrows indicate necrotic cells inside the tubular structures. The values are expressed as mean ± SD. Statistical analyses were performed as two-way ANOVA. The *p*-value is indicated in the graphs. Sham mice (filled circles); I/R, ischemia/reperfusion mice (filled squares); SDS-PAGE, sodium dodecyl sulfate polyacrylamide gel electrophoresis; G, glomerulus; PT, proximal tubule; TALH, thick ascending loop of Henle; CD, collecting duct; SD, standard deviation. Scale bar: 25 µm.

To evaluate the effects of apelin-13 on renal I/R, the mice were initially treated with apelin-13 (200 μg/kg/day) for 5 days and subjected to kidney bilateral I/R. With regard to renal function, renal I/R induced significant increases in the plasma urea levels in the vehicle + I/R group compared to the vehicle + sham group; moreover, pretreatment with apelin-13 did not attenuate the elevated plasma urea levels induced by renal I/R (Figure 2A; Table 2). The plasma creatinine levels remained comparable among the groups (Figure 2B; Table 2). In the vehicle + I/R group, the estimated creatinine clearance was markedly lower than that in the vehicle + sham group, and pretreatment with apelin-13 did not reduce the renal function impairment (Table 2). Albumin excretion was higher in the vehicle + I/R group, and this increase in albumin excretion induced by renal I/R was not attenuated by pretreatment with apelin-13 (Figures 2C, D; Table 2; Supplementary Figure S2). In terms of the metabolic parameters, mice in the apelin-13 + I/R group had lower food consumption than in the vehicle + I/R group (Table 2). Additionally, mice in the vehicle + I/R group demonstrated higher water consumption than the vehicle + sham group. Interestingly, the 24-h urine flow rates were comparable among the groups (Table 2). Furthermore, there were no significant differences in weight gain in the studied groups (Table 2). In the vehicle + I/R group, the ratio of right kidney weight to final bodyweight increased when compared to the vehicle + sham group, and pretreatment with apelin-13 did not attenuate this parameter in the apelin-13 + I/R group (Table 2). No significant changes were observed in plasma osmolarity in either the vehicle + I/R or apelin-13 + I/R groups (Table 2). In addition, pretreatment with apelin-13 significantly reduced the plasma osmolarity of the apelin-13 + sham group compared to the vehicle + sham group (Table 2).

**TABLE 2:** Statistical data of animal experimentation.

Parameter	Vehicle + Sham (n)	Vehicle + I/R (n)	Apelin-13 + Sham (n)	Apelin-13 + I/R (n)	Interaction Apelin-13 and I/R
Feed intake, g/day	6.429 ± 1.902 (7)	5.000 ± 1.414 (7)	5.333 ± 0.816 (6)	2.857 ± 0.690^&^ (7)	F (1, 23) = 1.06, P = 0.3133^ns^
Water intake, mL/day	1.571 ± 1.134 (7)	4.000 ± 2.582^*^ (7)	1.167 ± 0.753 (6)	2.714 ± 0.756 (7)	F (1, 23) = 0.556, P = 0.4636^ns^
Urinary flow, mL/24 h	1.680 ± 1.045 (7)	2.549 ± 1.669 (7)	1.299 ± 0.555 (6)	1.947 ± 0.463 (7)	F (1, 23) = 0.072, P = 0.7901^ns^
Weight gain, g	−2.857 ± 1.215 (7)	−3.857 ± 0.900 (7)	−2.833 ± 0.408 (6)	−3.571 ± 0.535 (7)	F (1, 23) = 0.163, P = 0.6902^ns^
Right kidney weight/final body weight, mg/g	6.840 ± 0.640 (7)	8.214 ± 0.796^**^ (7)	6.136 ± 0.312 (6)	8.278 ± 0.416 (7)	F (1, 23) = 2.92, P = 0.1008^ns^
Plasma urea, mg/dL	68.321 ± 12.281 (7)	167.917 ± 76.235^*^ (7)	59.105 ± 5.190 (6)	157.849 ± 71.877 (7)	F (1, 23) = 0.000419, P < 0.0001
Plasma creatinine, mg/dL	0.225 ± 0.060 (7)	0.423 ± 0.108 (7)	0.215 ± 0.046 (6)	0.525 ± 0.291 (7)	F (1, 23) = 0.794, P = 0.3821^ns^
Plasma osmolarity, mmol/kg	340.143 ± 23.348 (7)	337.143 ± 12.941 (7)	303.500 ± 17.886^#^ (6)	339.857 ± 23.184 (7)	F (1, 23) = 6.58, P = 0.0173
Creatinine clearance, mL/min	0.210 ± 0.093 (6)	0.083 ± 0.049^*^ (7)	0.195 ± 0.117 (6)	0.071 ± 0.025 (7)	F (1, 22) = 0.00229, P = 0.9623^ns^
Urinary albumin/creatinine, a.u	1,168.570 ± 447.156 (3)	36,968.220 ± 4,325.131^**^ (3)	2,553.372 ± 1,392.513 (3)	55,088.574 ± 14,755.191 (3)	F (1, 8) = 3.52, P = 0.0974 ^ns^
Tubular injury score, a.u	0.038 ± 0.052 (7)	2.675 ± 0.668^****^ (7)	0.056 ± 0.050 (6)	3.019 ± 0.473 (7)	F (1, 23) = 1.02, P = 0.3238^ns^
KIM-1 staining, a.u	1,542.857 ± 2,042.668 (7)	15,241.333 ± 6,754.069^****^ (6)	218.167 ± 534.397 (6)	11,138.286 ± 2,047.152 (7)	F (1, 22) = 0.981, P = 0.3328^ns^
NGAL staining, a.u	3,593.015 ± 4,194.488 (6)	20,291.000 ± 2,674.501^****^ (7)	2,000.727 ± 1,976.643 (6)	16,904.000 ± 2,593.302 (7)	F (1, 22) = 0.600, P = 0.4467^ns^
Megalin staining, a.u	44,272.571 ± 14,297.889 (7)	17,686.800 ± 7,606.102^**^ (5)	39,136.571 ± 14,348.401 (6)	22,251.286 ± 4,754.615 (7)	F (1, 22) = 1.16, P = 0.2923^ns^
Ki67 staining, cells/field	2.158 ± 1.240 (5)	89.707 ± 10.388^****^ (6)	0.533 ± 0.357 (6)	17.426 ± 11.176^&&&&^ (7)	F (1, 20) = 114, P < 0.0001
Phospho ERK 1/2 staining, a.u	3,449.667 ± 526.047 (6)	6,354.571 ± 1,881.148^**^ (7)	3,283.571 ± 931.567 (6)	2,557.714 ± 818.793^&&&&^ (7)	F (1, 23) = 16.0, P = 0.0006
IB of Phospho ERK 1/2, fold change	1.000 ± 0.000 (4)	1.594 ± 0.428^*^ (4)	1.171 ± 0.262 (4)	0.910 ± 0.153^&^ (4)	F (1, 12) = 10.6, P = 0.0068

The data were analyzed using two-way ANOVA and Tukey multiple comparisons test. ^*^ P < 0.05 versus vehicle + sham, ^**^ P < 0.01 versus vehicle + sham, ^****^ P < 0.0001 versus vehicle + sham. ^#^ P < 0.05 versus vehicle + sham. ^&^ P < 0.05 versus vehicle + I/R. ^&&&&^ P < 0.0001 versus vehicle + I/R. The values are given as mean ± SD. The number of animals (n) per group is indicated within parentheses. IB, immunoblotting. Ns, non-significant. A.u, arbitrary units.

**FIGURE 3:**
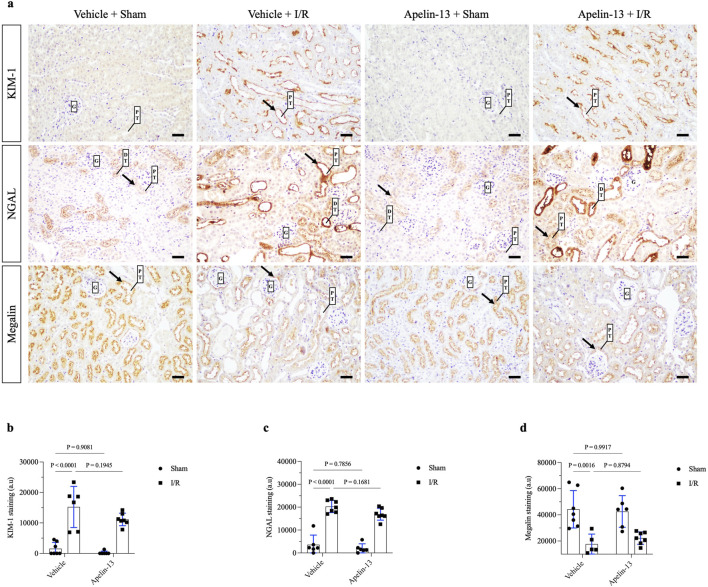
Exogenous apelin-13 administration sustains kidney tubular injury after renal I/R. **(A)** Representative immunohistochemistry from 20× micrographs (4 µm thick) of KIM-1, NGAL, and megalin and **(B, C, D)** graphical presentations showing that previous administration of exogenous apelin-13 sustained tubular injury markers KIM-1 and NGAL as well as decreased the levels of proximal tubular megalin in mice subjected to renal I/R. The black arrows indicate positive staining. The values are expressed as mean ± SD. Statistical analyses were performed as two-way ANOVA. The *p*-value is indicated in the graphs. Sham mice (filled circles); I/R, ischemia/reperfusion mice (filled squares); G, glomerulus; PT, proximal tubule; DT, distal tubule; SD, standard deviation. Scale bar: 25 µm.

Morphological analysis showed that renal I/R induced tubular swelling, cell necrosis, and proteinuric casts in the vehicle + I/R group compared to the vehicle + sham group (Figure 2E; Table 2). Similarly, exogenous apelin-13 failed to prevent kidney injury in the apelin-13 + I/R group compared to the vehicle + I/R group as the tubular swelling, necrotic cells, tubulointerstitial damage, and protein casts persisted from the cortex to the medulla (Figure 2E). These observations were consistent with elevated tubular injury scores (Figure 2F; Table 2). Tubular injury was characterized by IHC analysis using antibodies against the kidney injury molecule 1 (KIM-1) and neutrophil gelatinase-associated lipocalin (NGAL) markers. Our initial findings were supported by the observation that renal I/R significantly elevated tubular KIM-1 and NGAL markers in the vehicle + I/R group compared to the vehicle + sham group. Furthermore, pretreatment with apelin-13 did not alter the elevated levels of these injury markers (Figures 3A–C; Table 2).

Next, we evaluated the staining of the endocytic receptor megalin at the brush border of the proximal tubules. Renal I/R significantly diminished proximal megalin expression in the vehicle + I/R group compared to the vehicle + sham group. Moreover, the initial administration of apelin-13 did not prevent downregulation of megalin in the apelin-13 + I/R group compared to the vehicle + I/R group (Figures 3A, D; Table 2). Given that apelin-13 was not effective at preventing I/R-induced AKI, these findings prompted us to further investigate the tubular status, particularly in terms of cell proliferation.

### Exogenous apelin-13 administration reduces tubular cell proliferation and impairs ERK 1/2 phosphorylation subsequent to renal I/R

**FIGURE 4:**
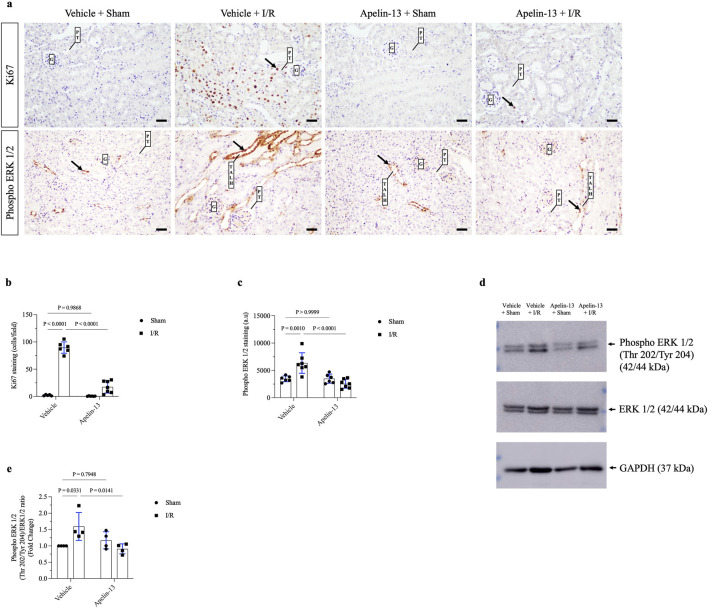
ERK 1/2-dependent tubular proliferation after renal I/R is impaired by apelin-13 administration. **(A)** Representative immunohistochemistry from 20× micrographs (4 µm thick) of Ki67 and phosphorylated ERK 1/2 and **(B, C)** graphical presentations showing that previous administration of exogenous apelin-13 decreased Ki67 and phospho ERK 1/2 staining in mice subjected to renal I/R. The black arrows indicate positive staining for each protein. **(D)** Representative immunoblotting of phosphorylated ERK 1/2, total ERK 1/2, and GAPDH, and **(E)** graphical presentation of the ratios. The values are expressed as mean ± SD. Statistical analyses were performed as two-way ANOVA. The *p*-value is indicated in the graphs. Sham mice (filled circles); I/R, ischemia/reperfusion mice (filled squares); G, glomerulus; PT, proximal tubule; TALH, thick ascending loop of Henle; SD, standard deviation. Scale bar: 25 µm.

We evaluated the proliferation marker Ki67 through IHC staining. Minimal staining of nuclear Ki67 was seen in the vehicle + sham or apelin-13 + sham group. As expected, renal I/R showed significantly increased Ki67 staining in the kidney cortex of the vehicle + I/R group compared to the vehicle + sham group. In contrast, pretreatment with apelin-13 attenuated the I/R-induced increase in tubular Ki67 staining (Figures 4A, B; Table 2). A significant interaction was observed between apelin-13 and I/R by two-way ANOVA (Table 2).

Next, we investigated ERK 1/2 signaling since MAPK contributes to tubular proliferation and regeneration in many tissues after ischemic injury (Kong et al., 2019). Based on immunohistochemistry, phospho ERK 1/2 (Thr202/Tyr204) was found to be upregulated in the tubular cells of the vehicle + I/R group compared to the vehicle + sham group. Similarly, immunoblotting showed that the ratio of phospho ERK 1/2 (Thr202/Tyr204) to total ERK 1/2 protein expressions was significantly higher in the vehicle + I/R group compared to the vehicle + sham group. Interestingly, this parameter was significantly lower in the apelin-13 + I/R group compared to the vehicle + I/R group (Figures 4A, C–E; Table 2; Supplementary Figure S2). A significant interaction was observed between apelin-13 and I/R by two-way ANOVA (Table 2). Taken together, these data suggest that pretreatment with apelin-13 reduces tubular proliferation upon renal I/R.

### PTCs after I/R or apelin-13 treatment show decreased intracellular pH recovery rate affecting Na^+^/H^+^ exchanger activity

**FIGURE 5:**
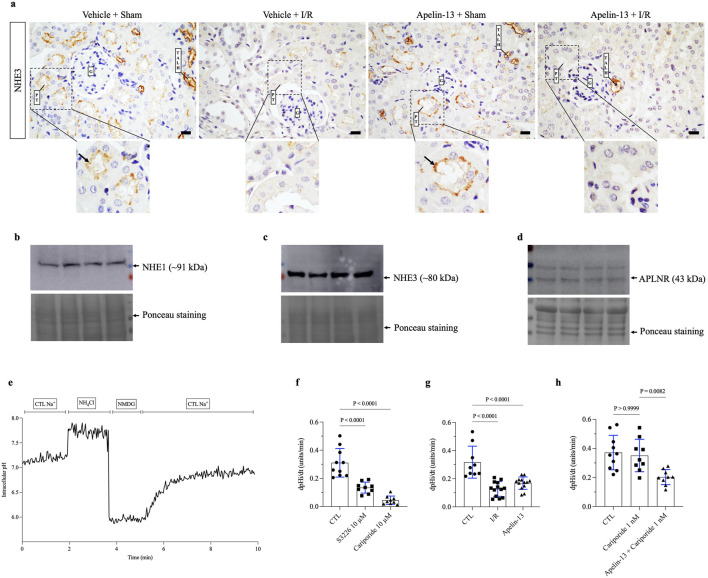
Both apelin-13 and I/R reduce intracellular pH recovery rates in the proximal tubular cells *in vitro*. **(A)** Representative immunohistochemistry from 40× micrographs (4 µm thick) showing that renal I/R affected NHE3 staining patterns in the proximal tubules of vehicle + I/R mice when compared to the sham group. Moreover, prior administration of exogenous apelin-13 did not affect the apical distribution of NHE3 in the proximal tubules adjacent to the glomerulus in the apelin-13 + sham group. The black arrows indicate positive staining for proximal tubular NHE3. **(B)** Protein expression of NHE1 in the TKPTS control cells. **(C)** Protein expression of NHE3 in the TKPTS control cells. **(D)** Protein expression of APLNR in the TKPTS control cells. **(E)** Representative experiment of pHi recovery in which the TKPTS cells were initially exposed to a ringer solution containing Na^+^ (138 mM), acid loading with NH_4_Cl (20 mM), a Na^+^-free solution with NMDG (138 mM), followed by a ringer solution containing Na^+^ (138 mM). **(F)** Effects of S3226 (10 µM) or cariporide (10 µM) on the pHi recovery rates of TKPTS cells. **(G)** Effects of I/R or apelin-13 (100 nM) on the pHi recovery rates of TKPTS cells. **(H)** Effects of cariporide (1 nM) or apelin-13 (100 nM) on the pHi recovery rates of TKPTS cells. The values are expressed as mean ± SD. Statistical analyses were performed as one-way ANOVA. The *p*-value is indicated in the graphs. I/R, ischemia/reperfusion; G, glomerulus; PT, proximal tubule; TALH, thick ascending loop of Henle; SD, standard deviation. Scale bar: 10 µm.

We investigated NHE3 activity based on the knowledge that renal I/R causes proximal tubular injury that directly impacts tubular transport. In a non-quantitative assessment, we evaluated NHE3 expression in mouse kidney tissue. The results indicated that apelin-13 administration did not affect the apical distribution of NHE3 in the proximal tubules or loop of Henle, showing a labeling pattern similar to that of the vehicle + sham group. However, in mice groups subjected to renal I/R, alterations were observed in the expression patterns of this transporter, particularly in the proximal tubules adjacent to the glomeruli (Figure 5A). To examine the effects of I/R and apelin-13 on NHE3 activity *in vitro*, we first confirmed that the TKPTS cells express NHE1, NHE3, and APLNR (Figures 5B–D; Supplementary Figure S3). A representative trace of pHi recovery showed that introduction of a Na^+^ control solution induced pHi recovery after acid loading (Figure 5E). It should be noted that the TKPTS cells are Na^+^-dependent on pHi recovery rate after acid loading.

Inhibition of NHE3 with S3226 significantly reduced the pHi recovery rate following acid loading compared to the control (CTL) cells (Figure 5F; Table 3). Similarly, a high dose of cariporide (10 µM) inhibited NHE1 and NHE3 activities, which sharply reduced the pHi recovery rate (Figure 5G). In addition, *in vitro* I/R significantly reduced the pHi recovery rate after acid loading equivalent to that of apelin-13 compared to the CTL group (Figure 5H; Table 3).

**TABLE 3:** Statistical data of cell pHi experiments.

Parameter	CTL (n = 9)	I/R (n = 13)	Apelin-13 (n = 13)
dpHi/dt, units/min	0.317 ± 0.114	0.127 ± 0.052^****^	0.168 ± 0.044^****^

Parameter	CTL (n = 10)	S3226 10 µM (n = 9)	Cariporide 10 µM (n = 9)
dpHi/dt, units/min	0.311 ± 0.102	0.134 ± 0.039^****^	0.044 ± 0.029^****^

Parameter	CTL (n = 10)	Cariporide 1 nM (n = 9)	Apelin-13 + Cariporide 1 nM (n = 9)
dpHi/dt, units/min	0.372 ± 0.119	0.351 ± 0.112	0.203 ± 0.051^##^

The data were analyzed using one-way ANOVA and Bonferroni multiple comparisons test. ^****^ P < 0.0001 versus CTL cells. ^##^ P < 0.01 versus cariporide 1 nM treated cells. The values are given as mean ± SD. The number of coverslips (n) per group is indicated within parentheses.

To specifically evaluate the effects of apelin-13 on NHE3 activity, we used cariporide at a low concentration (1 nM) to selectively inhibit NHE1. In the CTL cells, the pHi recovery rate was 0.372 ± 0.119 pHi units/min. This recovery rate was not significantly reduced by NHE1 inhibition with 1 nM of cariporide (0.351 ± 0.112 pH units/min) compared to the CTL cells (Figure 5G; Table 3). However, the results showed that apelin-13 significantly reduced the pHi recovery mediated by the cariporide-resistant NHE3 isoform (0.203 ± 0.051 pH units/min) by 42% (Figure 5G; Table 3). These findings indicate that apelin-13 treatment significantly diminishes the Na^+^-dependent pHi recovery rate after cell acidification, primarily through inhibition of NHE3 activity in the TKPTS cells.

## Discussion

The renal I/R-induced AKI model is characterized by temporary obstruction of blood flow to the kidneys followed by reoxygenation (Sharfuddin and Molitoris, 2011). All kidney compartments are affected in this condition, including the glomeruli and endothelium (de Ponte et al., 2021; Sharfuddin and Molitoris, 2011). Low ATP availably to the tubular cells causes cell dysfunction as the metabolic demand is high (Munshi et al., 2011). Indeed, cells can detach from the basal membrane, especially through cytoskeleton alterations and loss of polarity as well as cell junctions, whereas the remaining tubular cells are subjected to apoptosis, necrosis (Sharfuddin and Molitoris, 2011), and more recently necroptosis, ferroptosis, and pyroptosis (Li et al., 2024a). In this condition, endothelial cell apoptosis, pericyte migration, and capillary rarefaction facilitate AKI-to-CKD progression (Kwiatkowska et al., 2023). Although there is no effective preventive method, the quest for safe therapeutic options that can minimize the consequences of renal I/R holds great clinical relevance.

In the initial experiments, downregulation of apelin mRNA expression was detected after renal I/R compared to the sham group, which was consistent with data from literature (Liu et al., 2023; Chen et al., 2015). This low gene expression of apelin was counteracted by upregulation of APLNR in the tubules of I/R mice, suggesting a feedback loop. The apelinergic system is involved in the immediate response to AKI (Patil et al., 2024; Chapman et al., 2021). Downregulation of this peptide has been reported in a model of unilateral renal I/R injury as well as in contrast-induced AKI (Liu et al., 2023; Chen et al., 2015). Thus, we firmly believe that the decrease in endogenous gene expression of apelin could be a consequence of cell injury, decreased tubular viability, or epigenetic modifications and that such effects somehow limit apelin signaling even with increased expression of its receptor.

We then evaluated whether a high dose of apelin-13 (200 μg/kg/day) would be beneficial for attenuating kidney injury in the I/R-induced AKI model. Consequently, the results showed that apelin-13 did not alter the deleterious effects of renal I/R, as evidenced by all the renal I/R characteristics, including low creatinine clearance, high plasma urea level, and albuminuria. In terms of the metabolic parameters, apelin-13 was responsible for diminished feed intake in the apelin-13 + I/R group. Similarly, apelin-13 has been shown to reduce feed intake in rats when administered intracerebroventricularly (1–2 µg/rat), likely owing to the presence of APLNR in the hypothalamus, where it regulates appetite (Ferrante et al., 2016). Under heightened inflammation conditions, such as obesity or diabetes, apelin-13 at a concentration of 20 nM may also influence the hypothalamic regions in mice, leading to the production of ROS (Drougard et al., 2014). However, in the absence of such injury, the effects of apelin-13 on these regions may be more limited. Interestingly, apelin-13 also reduced the plasma osmolarity in the apelin-13 + sham mice group; this finding is in line with previous observations where apelin-13 was found to inhibit vasopressin secretion and act in the distal nephron, hindering V2 receptor signaling, AQP2 apical insertion, and ENaC activity as well as inducing diuresis (Ayari and Chraibi, 2022; Chen et al., 2021; Boulkeroua et al., 2019).

Compared to the vehicle + I/R group, apelin-13 administration in the apelin-13 + I/R group neither attenuated the tubular injury markers KIM-1 and NGAL nor improved megalin expression at the brush border of the PTCs. Previous studies have shown that KIM-1 and NGAL are not only the markers of early kidney injury but also signaling glycoproteins involved in inflammation, cell-cycle arrest, and mitochondrial dysfunction (Chen et al., 2023; Marques et al., 2023; Mori et al., 2021). These findings are significant because the persistent upregulation of KIM-1 and NGAL chronically could be relevant to renal fibrosis as well as AKI-to-CKD progression (Dong et al., 2019; Humphreys et al., 2013; Bonventre and Yang, 2011). Megalin is an endocytic receptor involved in the internalization of several molecules in PTCs, especially albumin (Nielsen et al., 2016). In line with the initial findings of albuminuria, apelin-13 was ineffective at protecting the PTCs from megalin downregulation induced by renal I/R.

With respect to tubular proliferation, exogenous administration of apelin-13 blocked renal I/R-induced increase in Ki67 nuclear staining and phosphorylation of tubular ERK 1/2. The signaling cascade of ERK 1/2 was previously described in renal I/R by Hu et al. (2021a), who observed that the activation of epidermal growth factor receptor (EGFR) signaling in the renal I/R mouse model was linked with phosphorylation of AKT (Ser473) and ERK 1/2 (Thr202/Tyr204), resulting in increased tubular Ki67 marker levels after ischemic insult; this indicates that that ERK 1/2 activation is directly associated with tubular recovery. In this sense, inhibition of ERK 1/2 phosphorylation is associated with reduced tubular proliferation, cell polarity, and profibrotic signals, thereby sustaining kidney injury (Hu et al., 2021a; Jang et al., 2013; Masaki et al., 2003). The results suggest that apelin-13 could interfere with cellular regeneration after ischemia-induced AKI, switching the adaptive repair mechanism to maladaptive response by downregulating tubular mitosis. Moreover, prior administration of apelin-13 to the sham animals did not affect any of these parameters, thus supporting the interactions between renal I/R and apelin-13.

Most studies in literature regarding the effects of apelin-13 in experimental models of kidney disease report a protective role of the peptide that prevents renal injury. In renal I/R models, effective doses were reported to range from as low as 5 μg/kg/day to as high as 30 μg/kg/day (Zheng et al., 2025; Chen et al., 2015). However, in diabetic mice, exogenous apelin-13 administrations at a dose of 30 μg/kg/day exacerbated podocyte effacement, endothelial dysfunction, endoplasmic reticulum stress, and impaired autophagy in the kidneys (Liu et al., 2017b; Guo et al., 2015). In this study, a higher dose of apelin-13 was administered (Li et al., 2018), which may have offset its potential renoprotective effects. These findings indicate the possibility of a dose-dependent biphasic effect of apelin-13, where higher concentrations may not yield protective outcomes.

Recent clinical studies have identified apelin-13 as a potential biomarker in patients with CKD, suggesting a compensatory increase in response to renal impairment and cardiovascular disturbance regardless of the presence of diabetes (Sakran et al., 2025; Wang et al., 2024; Nyimanu et al., 2022). Additionally, acute administration of pyroglutamate apelin-13 to CKD patients was shown to enhance renal blood flow, induce a mild reduction in blood pressure, decrease proteinuria, and promote sodium excretion, all of which could influence the progression of kidney disease (Chapman et al., 2024). The study of the apelinergic system in human AKI is an emerging and promising area of research; however, further clinical and translational studies are required to clearly define its role as a biomarker or therapeutic target.

The downregulation of SLC proteins may impair renal function recovery following ischemia-induced AKI (Faucher et al., 2020). The present study is a pioneering effort at demonstrating that apelin-13 reduces the activity of proximal tubular NHE3. Previous findings indicate that apelin-13 binds to APLNR and activates the Gi or Gq protein, which subsequently stimulates protein kinase C (PKC) to directly enhance NHE1 activity in cardiomyocytes (Magalhães et al., 2016). However, phosphorylation of NHE3 at the serine residues by PKC is considered a regulatory mechanism that could indirectly reduce its activity (Kocinsky et al., 2007; Wiederkehr et al., 1999). Additionally, *in vitro* models of cellular ischemia demonstrate that ATP depletion impacts the energy required to maintain active tubular transport processes (Zhao et al., 2004). *In vivo*, the changes in the apical distribution of NHE3 staining observed after renal I/R appear to be the result of a multifactorial process that includes oxidative stress, inflammation, and metabolic disruptions. The lack of protective effects of apelin-13 is in agreement with our findings, suggesting that the apelin/APLNR signaling pathway may be either disrupted or insufficient to counteract the deleterious effects of ischemia-induced AKI. As a limitation of this study, exploring alternative approaches with varying doses or administration frequencies of apelin-13 could help determine whether the lack of a protective effect is associated with the therapeutic regimen. Thus, the relevant questions warrant further investigations.

Considered together, the above results indicated that although apelin-13 did not attenuate tubular injury, the reduced levels of endogenous apelin mRNA after renal I/R could be important for carefully adjusting the pharmacological modulation of this pathway. Considerable caution is required in this regard because apelin-13 administration at high doses can affect tubular proliferation, delay tissue recovery, and possibly increase the severity of ischemia-induced AKI.

## Data Availability

The data presented in the study are deposited in the Figshare repository, accession number 10.6084/m9.figshare.28555712 (https://figshare.com/articles/dataset/dx_doi_org_10_6084_m9_figshare_28555712/28555712).
