# Supplementary material for: Insights into the effects of apelin-13 on renal function and NHE3 activity following ischemia/reperfusion-induced acute kidney injury
Source: Front Physiol. 2025 Mar 19;16:1544274. doi: 10.3389/fphys.2025.1544274 (PMC11961903; doi:10.3389/fphys.2025.1544274)
Supplement: Supplementary file 1 [file Presentation1.pdf]

Insights into the apelin-13 effects on renal function and NHE3 activity following ischemia/reperfusion-induced acute kidney injury

Guilherme Lopes-Gonçalves[1*], Juliana Martins Costa-Pessoa[1], Mariana Charleaux de Ponte[2], Heitor Macedo Braz[1], Maria Oliveira-Souza[1*]

[1]*Laboratory of Renal Physiology, Department of Physiology and Biophysics, Institute of Biomedical Sciences, University of Sao Paulo, Sao Paulo, Brazil.*

[2]*Laboratory of Cellular and Molecular Bases of Renal Physiology, Department of Physiology and Biophysics, Institute of Biomedical Sciences, University of Sao Paulo, Sao Paulo, Brazil.*

Supplementary Information

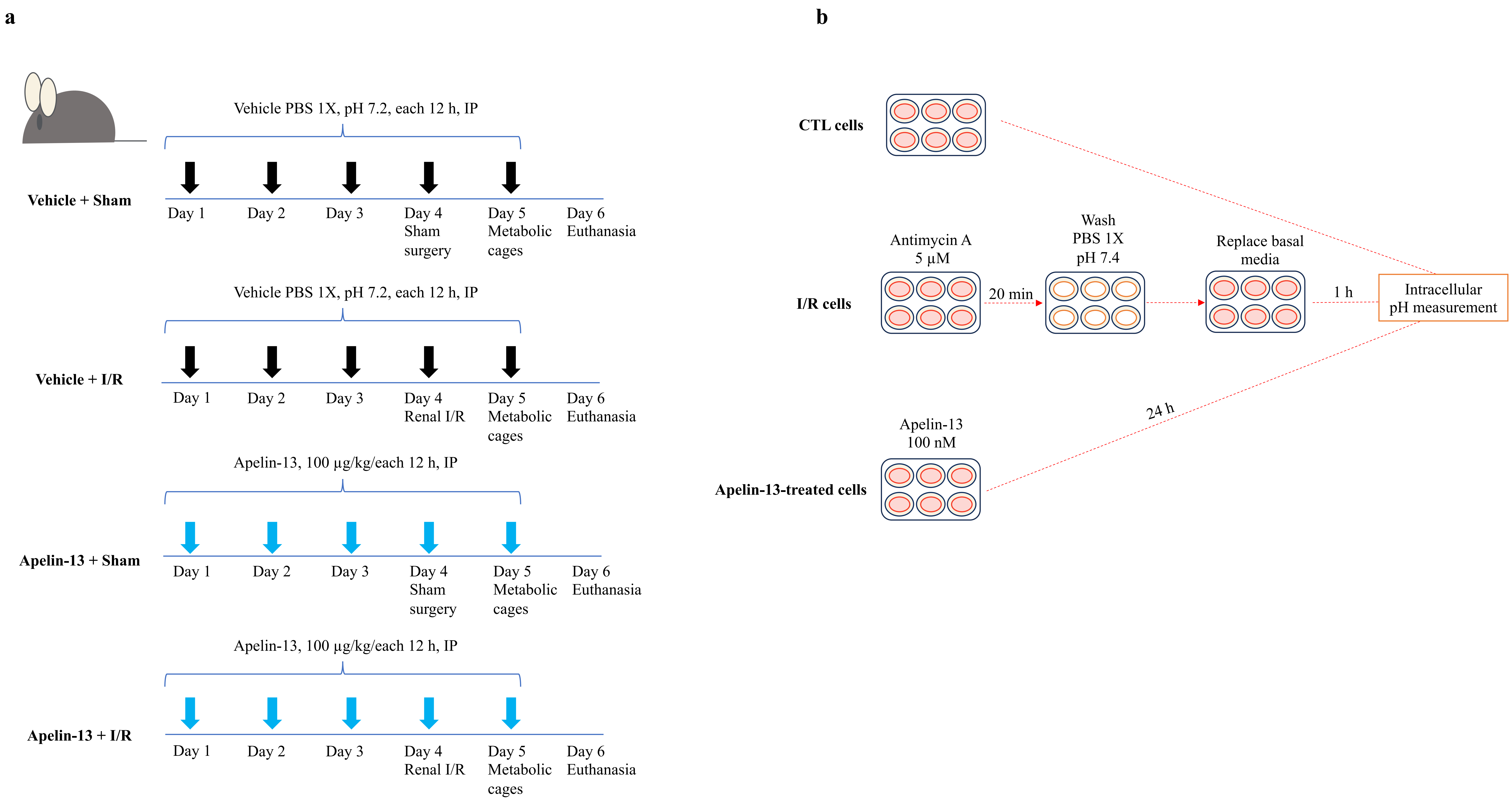

Supplementary Figure S1. Experimental design. (a) Experimental model of renal I/R in C57BL/6 mice. Black arrows represent vehicle (PBS 1X, pH 7.2) administrations, whereas blue arrows represent apelin-13 (100 µg/kg/each 12 h, corresponding to 200 µg/kg/day) IP administrations. (b) *In vitro* experiments with TKPTS cells. Cells were treated with antimycin A (5 µM, Sigma-Aldrich) diluted in DMEM flex media without glucose, pyruvate or amino acids (A2493901, Thermo Fisher, Waltham, MA, USA) for 20 min to induce ischemia with ATP depletion. After one wash with 1X PBS, reperfusion was made with basal media for 1 h. All steps of pHi recovery for apelin-13 treatment were performed using solutions containing 100 nM apelin-13 (Cayman Chemicals). To evaluate NHE3 contribution in pHi recovery rate, cells were treated with 10 µM S3226 (Sigma-Aldrich) or 10 µM Cariporide (Santa Cruz Biotechnology) at pHi recovery phase. To evaluate NHE1 contribution in pHi recovery rate, cells were treated with 1 nM Cariporide (Santa Cruz Biotechnology) at pHi recovery phase. For all experiments, the initial pHi recovery rate was calculated (dpHi/dt, pH units/min) during the first two minutes of the recovery phase using linear regression analysis. Sham, sham surgery; I/R, ischemia/reperfusion; IP, intraperitoneal.

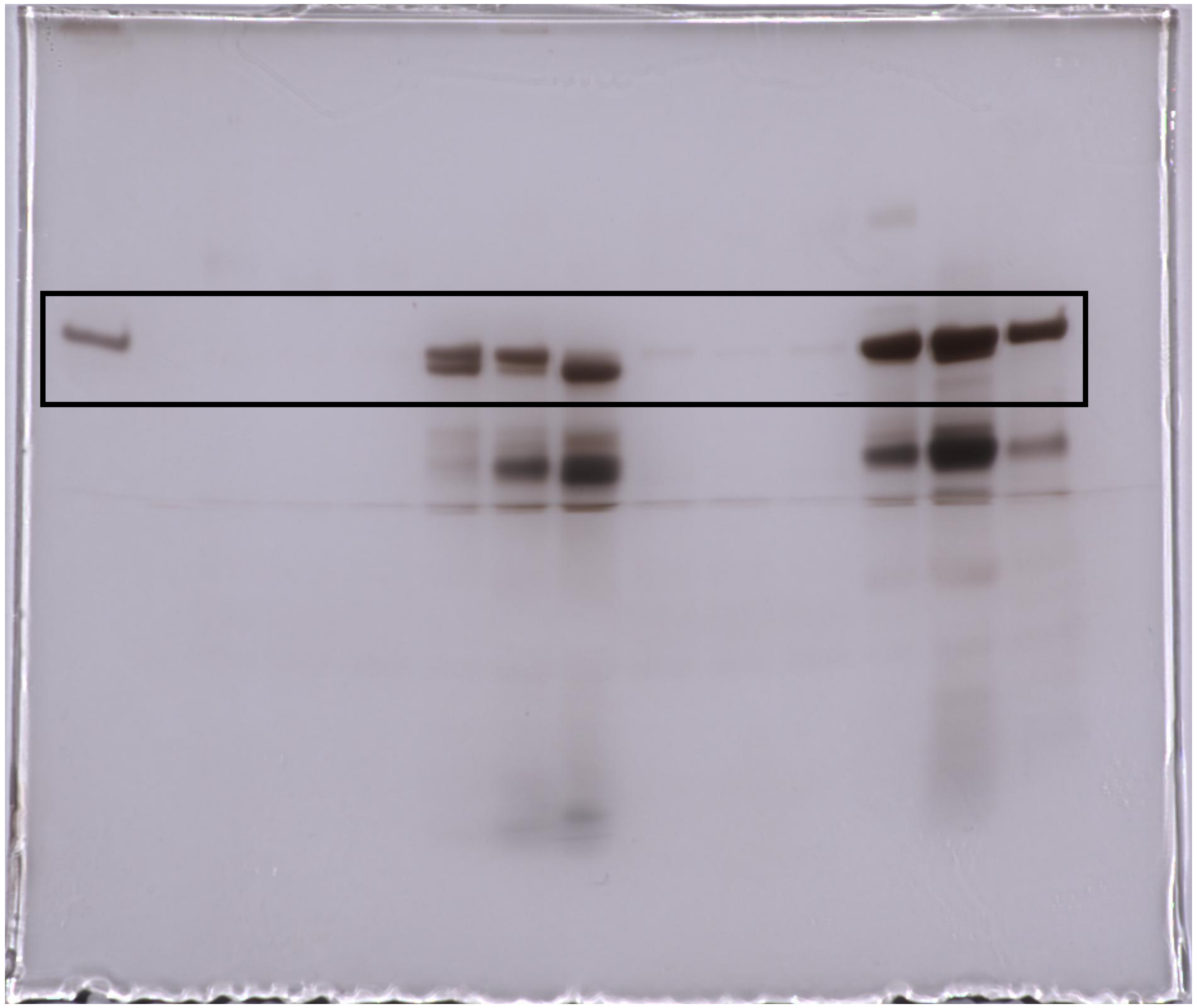

BSA
~66 kDa

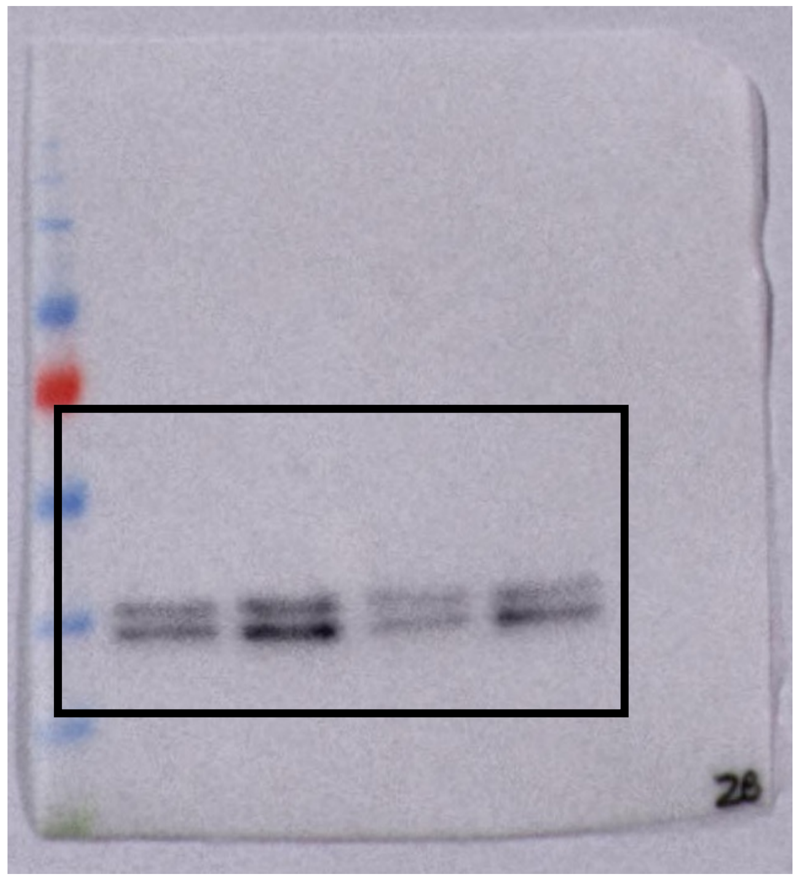

Phospho ERK 1/2
(Thr 202/Tyr 204)
(42/44 kDa)

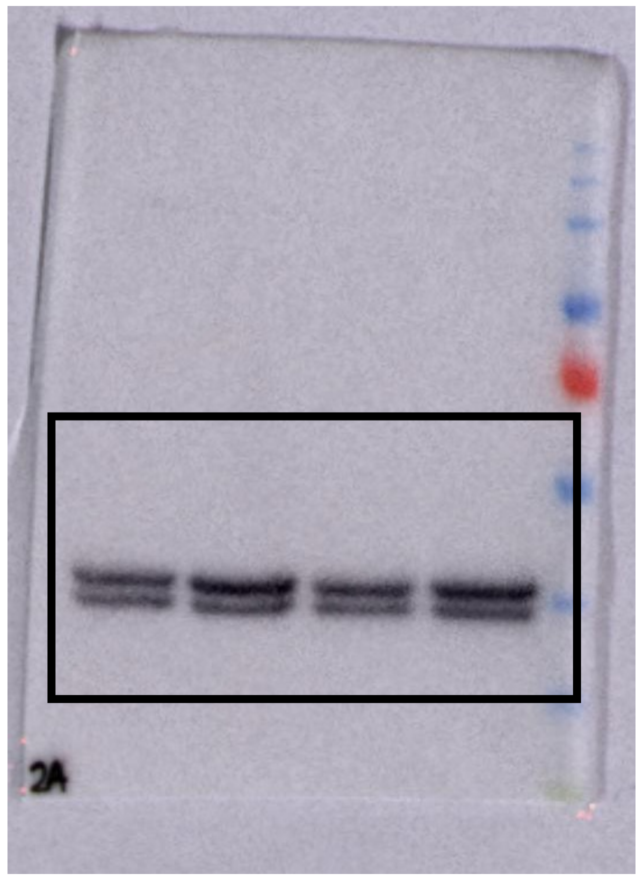

ERK 1/2
(42/44 kDa)

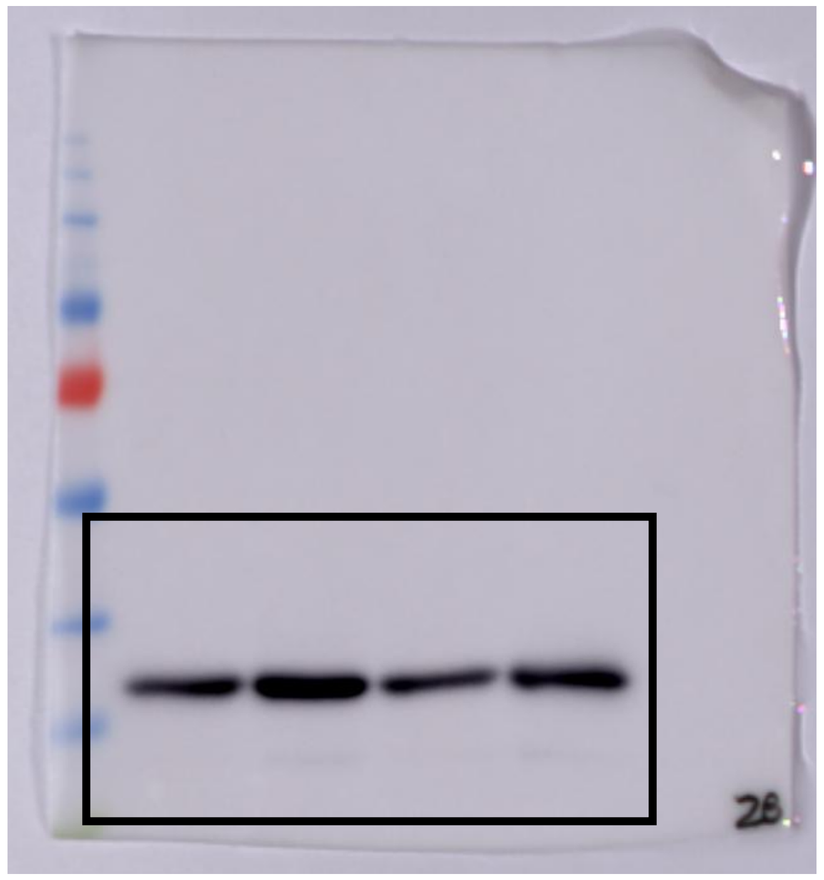

GAPDH
(37 kDa)

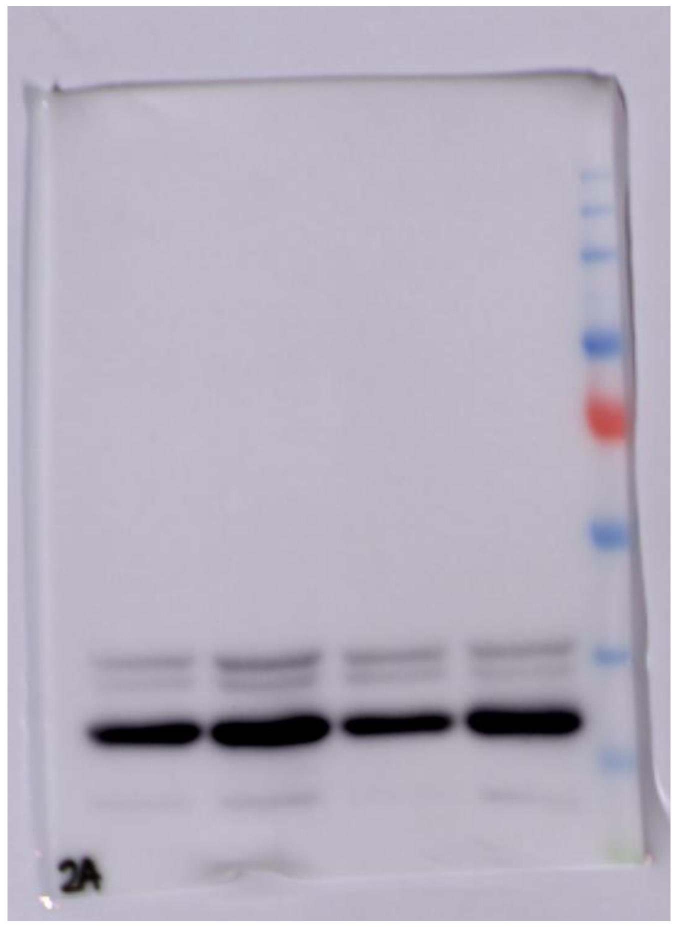

GAPDH
(37 kDa)

Supplementary Figure S2. Unedited gels of 24-hour urine and phospho ERK 1/2, ERK 1/2 and GAPDH immunoblotting experiments.

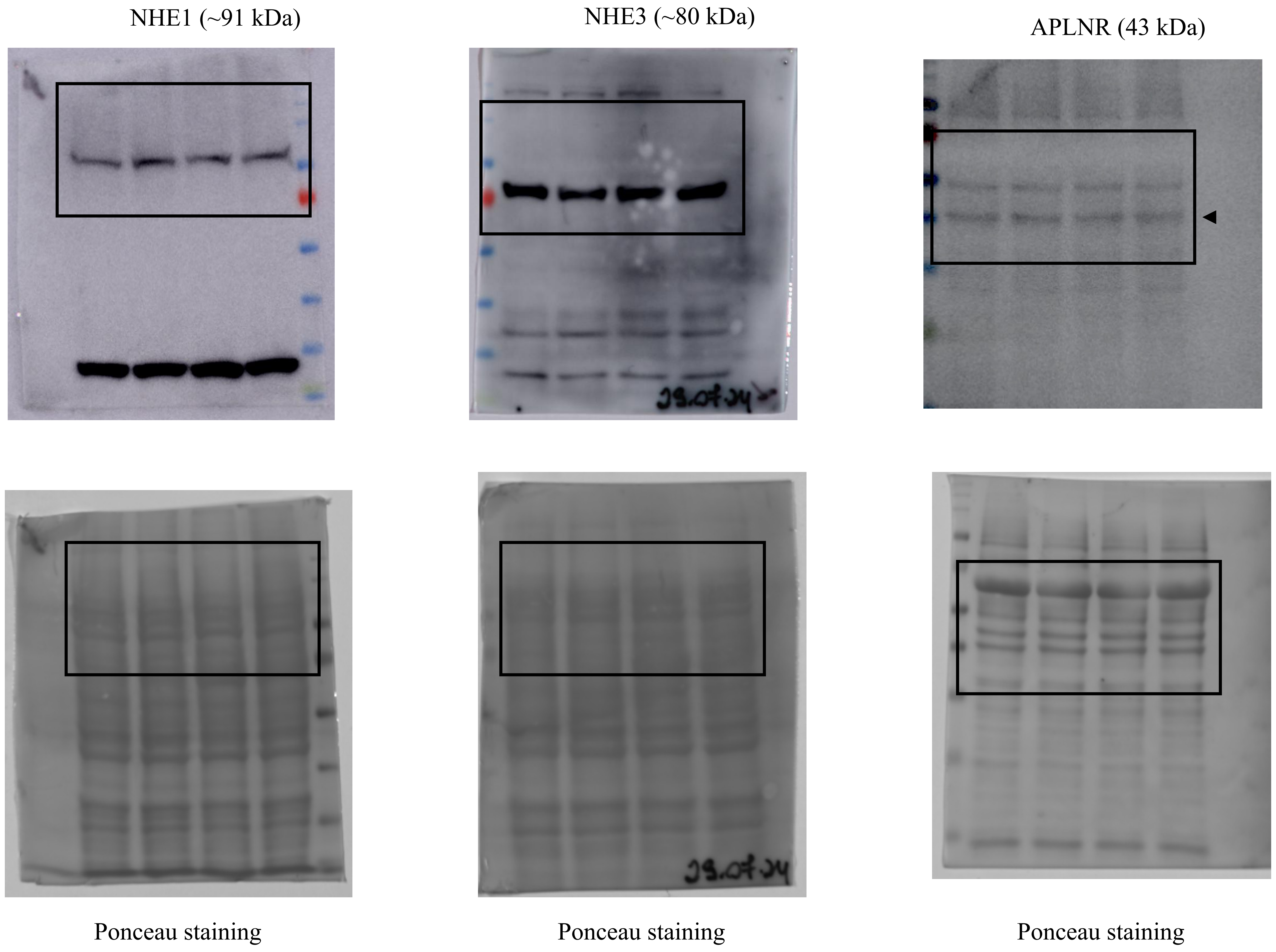

Supplementary Figure S3. Unedited gels of NHE1, NHE3 and APLNR immunoblotting experiments.

Supplementary Methods. Quantification of immunohistochemistry experiments.

KIM1 immunohistochemistry quantification. Fifteen 20x cortical and cortical medullary areas were opened in ImageJ software (National Institutes of Health, Bethesda, MD, USA). We used the plugin color deconvolution. Next, DAB images were submitted to threshold (min: 0, max: 160). The mean values were used for statistical analysis.

APLNR immunohistochemistry quantification. Fifteen 20x cortical and cortical medullary areas were opened in ImageJ software (National Institutes of Health, Bethesda, MD, USA). We used the plugin color deconvolution. Next, DAB images were submitted to threshold (min: 30, max: 80). The mean values were used for statistical analysis.

NGAL immunohistochemistry quantification. Fifteen 20x cortical and cortical medullary areas were opened in ImageJ software (National Institutes of Health, Bethesda, MD, USA). We used the plugin color deconvolution. Next, DAB images were submitted to threshold (min: 0, max: 100). The mean values were used for statistical analysis.

Megalin immunohistochemistry quantification. Fifteen 20x cortical and cortical medullary areas were opened in ImageJ software (National Institutes of Health, Bethesda, MD, USA). We used the plugin color deconvolution. Next, DAB images were submitted to threshold (min: 0, max: 170). The mean values were used for statistical analysis.

Phospho ERK1/2 immunohistochemistry quantification. Fifteen 20x cortical and cortical medullary areas were opened in ImageJ software (National Institutes of Health, Bethesda, MD, USA). We used the plugin color deconvolution. Next, DAB images were submitted to threshold (min: 0, max: 95). The mean values were used for statistical analysis.

Ki67 immunohistochemistry quantification. Fifteen 20x cortical and cortical medullary areas were opened in ImageJ software (National Institutes of Health, Bethesda, MD, USA). Ki67 positive cells were manually counted. The mean values were used for statistical analysis.
